# Current state of NK cell-mediated immunotherapy in chronic lymphocytic leukemia

**DOI:** 10.3389/fonc.2022.1077436

**Published:** 2023-01-04

**Authors:** Zong-Han Wang, Wei Li, Hao Dong, Fujun Han

**Affiliations:** ^1^ Cancer Center, The First Hospital of Jilin University, Changchun, Jilin, China; ^2^ Department of General Surgery, Second Affiliated Hospital of Jilin University, Changchun, Jilin, China; ^3^ Department of Gastrointestinal Nutrition and Surgical Surgery, The Second Affiliated Hospital of Jilin University, Changchun, Jilin, China

**Keywords:** CLL, NK cells, immunotherapy, receptors, anti-tumor activity

## Abstract

Chronic lymphocytic leukemia (CLL) has become one of the most common hematological diseases in western countries, with an annual incidence of 42/100,000. Conventional chemotherapy and targeted therapeutic drugs showed limitations in prognosis or in efficiency in high-risk patients. Immunotherapy represented is one of the most effective therapeutic approaches with the potential of better effect and prognosis. Natural killer (NK) cells are good options for immunotherapy as they can effectively mediate anti-tumor activity of immune system by expressing activating and inhibiting receptors and recognizing specific ligands on various tumor cells. NK cells are critical in the immunotherapy of CLL by enhancing self-mediated antibody-dependent cytotoxicity (ADCC), allogeneic NK cell therapy and chimeric antigen receptor-natural killer (CAR-NK) cell therapy. In this article, we reviewed the features, working mechanisms, and receptors of NK cells, and the available evidence of the advantages and disadvantages of NK cell-based immunotherapies, and put forward future study directions in this field.

## Introduction

Chronic lymphocytic leukemia (CLL) has become one of the most common hematological diseases in western countries, with an annual incidence of 42/100,000. Its incidence rate has reached 30/100000/year in people over 80 years old (1). Starting with the standard treatment methods based on chemotherapy, including purine analogues and alkylating agents, the treatment methods for patients with CLL are also constantly innovating and developing recently (2, 3). Despite the strong antitumor activity of conventional chemotherapy, there was no improvement in overall survival (OS) in patients with CLL (4). Recently, the emergence and vigorous development of targeted therapies, including B-cell Lymphoma 2 (Bcl-2) protein inhibitors and B-cell receptor (BCR) signal inhibitors have greatly improved the treatment options and prognosis of CLL (5). Although the effectiveness of the above targeted therapeutic drugs has been widely confirmed, it still shows some obvious potential limitations, including poor treatment efficiency in high-risk patients and the occurrence of drug resistance (6, 7). In such cases, the introduction of other novel therapies to achieve better treatment response and reduce the occurrence of drug resistance becomes a necessity. Immunotherapy is one of the most effective therapeutic approaches.

A series of new immunotherapy methods, including immune checkpoint blocking and chimeric antigen receptor (CAR) transduction, have shown significant therapeutic effects and application prospects in a variety of lymphoid malignancies (8, 9), but the results in CLL (10, 11) cannot meet the expectations, which may be caused by the defect of effector T cells (12, 13). Therefore, further exploring the therapeutic effects of other types of immune cells in CLL can make the immunotherapy of CLL clearer and more complete. There are specific ligands on the surface of tumor cells, and activated receptors and inhibited receptors expressed by natural killer cells (NK) can specifically bind to the former, thus mediating the anti-tumor activity of immune system (14–16). Antibody-dependent cytotoxicity is an important mechanism by which NK cells exert the effects of killing cancer cells, which is mediated by CD16 receptors (17). According to the characteristics and functional characteristics of NK cells, this paper discussed the latest research progress of CLL immunotherapy with NK cells as the core.

## Overall view on NK cells

As an important part of the natural immune system, NK cells play a key role in microbial infection and tumor cell recognition. Generally, NK cells are classified according to the difference of CD 56 density expression, that is, CD3^−^CD56^bright^CD16^−^ NK cells and CD3^−^CD56^dim^CD16^+^ NK cells (18, 19). Mainly existed in the peripheral blood system, CD3^−^CD56^dim^CD16^+^ NK cells are associated with cytolytic activity by secreting granzymes and perforin into target tumor cells (19). On the contrary, CD3^−^CD56^bright^CD16^−^ NK cells generally exist in secondary lymphoid tissues, where they express and release various cytokines to fight microbial infection or cancer cell proliferation (19, 20). It has been demonstrated that CD56^bright^ NK cells may differentiate into CD56^dim^ NK cells with the stimulation of peripheral tissue fibroblasts (21).

### Role of NK cells in the immune system

NK cell activity is controlled by the balance between various activation, inhibition and cytokine receptors expressed on the cell surface, which bind to specific ligands expressed on the surface of immune cells, host cells and tumor cells (22–24). When NK cells contact with normal immune cells or host tissue cells, the inhibitory receptor ligand signaling pathway and MHC-I specific ITIM-bearing receptors will play a prominent role and make NK cell toxic activity in a resting state (22, 25). When NK cells sense the cancer cells or microbial infection in the environment, their activated receptor ligand interaction signal pathway will play a leading role and they will release cytokines and lytic granules to damage the activity of target cells (26). Since NK cells can only be activated to produce key apoptosis-related cytokines in the presence of multiple interactions, this ensures that NK cells do not accidentally injure healthy tissues and cells on a large scale under normal circumstances.

NK cells mainly perform two kinds of functions in tumor cells: transmitting signals to circulating immune cells by releasing cytokines; inducing the lysis apoptosis of tumor cells by degranulation. Cytokines released by NK cells including GM-CSF, IFN-γ, IL-33 and TNF-α, combined with IL-4, IL-7, and IL-12 stimulate the recruitment and activity of hematopoietic cells to enhance the immune response (27). The involvement of NK cells in cytolytic killing of tumor cells include direct killing through releasing perforin and granzyme and ADCC (28–30). In ADCC, the Fc receptors including FcγRIIIA and/or FcγRIIC expressed on the surface of NK cells can bind to the Fc portion of IgG1 or IgG3 whose Fab portion bound to tumor cells and the NK cells get activated as a result. Then NK cells can release lytic granules to kill the targeted tumor cells without pre-activation (31). Some of the NK cell receptors are already found to be valuable for leukemia treatment and tumor surveillance, which were PD-1, 2B4 (CD244), CS1(CD319), LLT1 (CLEC2D) that regulate NK cell cytotoxicity, and NKp44, NKp30, and NKp46 known as natural cytotoxicity receptors (NCRs).

### Natural killer cell receptors

The functioning of NK cells is controlled by the activating and inhibitory signals transmitted from the target cells which are presented as the binding of receptors and ligands (16, 32).

### Activating receptors

**Figure 1:**
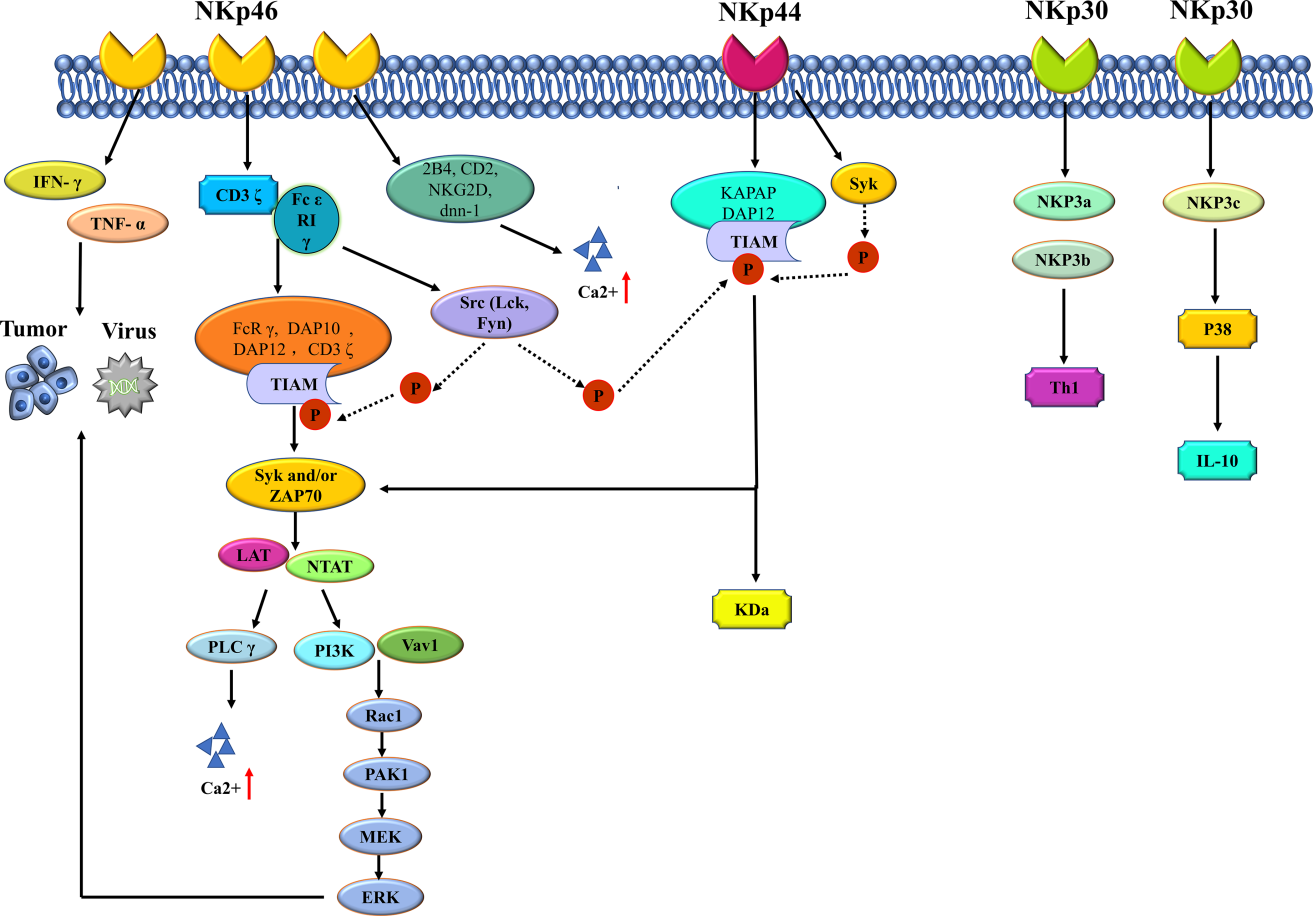
The mechanism of NCR protein of NK cell.

The main type of activating receptors of NK cells are the NCRs, which belong to immunoglobulin-like family. NCRs regulates the recognition of viruses, bacteria and cancer cells by the immune system (31). Members of this protein family are important in immune recognition, including NKp 46 (NCR 1, CD 335), NKp 44 (NCR 2, CD 336) and NKp 30 (NCR 3, CD 337) (33). The mechanism of NCR protein is shown in **Figure 1**. There is a gene encoding NKp 46 protein on human chromosome 19. Intracellular NKp 46 protein does not contain ITAM sequence, and the amino acid residues in the transmembrane region of NKp 46 protein act as the linker of ϵ RI γ and CD 3 ζ, which plays a role in transmitting activation signals (34). The ligands recognized by NKp 46 mainly include four categories: tumor cell ligands, viral ligands, bacterial ligands and parasite ligands (35). The most classical tumor ligands are those of melanoma and myeloma, while the ligands of most tumor cells are still unknown. Hemagglutinin (HA) and hemagglutinin neuraminidase (HN) are common virus ligands of NKp 46. When the cells are infected by Mycobacterium tuberculosis, vimentin appears on the cell surface, which is the bacterial ligand of NKp 46. The erythrocyte membrane protein of plasmodium falciparum (PfEMP 1) belongs to the parasitic ligand of NKp 46. NK46 protein is expressed in all mature NK cells and is responsible for initiating the killing process of NK cells. The expression level of NKp 46 indicates the cytotoxicity of NK cells. When NKp 46 protein binds to its ligand, the killing program of NK cells is activated, and the levels of IFN-γ and TNF-α are increased, thus exerting the anti-infection immunity and killing tumor cells (36). The activation signal of NK cells mainly depends on ITAM-related receptors, which need activation of NKp 46 protein. This cascade signaling is ubiquitous in NK cells. The junction proteins of DAP 12, FcRγ, DAP 10 and CD 3 ζ all contain ITAM, while NK cells can express type I transmembrane proteins Fcϵ RIγ, CD 3 ζ and DAP 12. When NKp 46 protein binds to the ligand, CD 3 ζ and Fc ϵ R 1 γ will also bind to NKp 46 protein, accompanied by the phosphorylation of ITAM in the linker protein, which is closely related to Src family kinases such as Lck and Fyn (35, 37). When ITAM in the linker protein is phosphorylated, tyrosine kinases such as Syk and/or ZAP 70 will be recruited and activated, which requires the SH 2 domain to play a major role (38). When tyrosine kinases are recruited and activated, transmembrane linker proteins including LAT and NTAT will also be activated, thus activating downstream phospholipase C (PLCγ), phosphatidylinositol - 3 - hydroxy kinase (PI 3K) and Vav 1, Vav 2 and Vav 3. When PLCγ is activated, the influx of extracellular calcium ions is enhanced. After activation of PI 3K and Vav 1, small G protein Rac 1 is recruited, and then cascade phosphorylation is induced through PAK1-MEKEERK signaling pathway, thus activating MAPK signaling pathway (39). Signal cascade reaction causes a series of gene expression promotion, including actin cytoskeleton rearrangement, degranulation, cytotoxicity and cytokines or chemokines. NKp46 protein plays a synergistic role with other commonly activated receptors in the process of activating cytotoxicity of NK cells. Relevant studies show that the Ca2+ influx of NK cells is closely related to the activation signal transmission process related to NKp46, in which the binding proteins of NKp46 are 2B4, CD2, NKG2D and DNAM-1 (40). However, whether the synergistic effect of NKp46 protein and different types of co-activated receptors is the same still needs further study.

The gene encoding the NKp30 protein and the NKp40 protein is present on human chromosome 6. There are three main specific ligands for NKp30 protein, which are tumor cell ligands, partial virus ligands, and parasitic ligands. B7-H6, BAG6/BAT3 and galectin -3 belong to the common tumor cell ligands of NKp30 protein. Viral ligands of the protein include vaccinia virus, human cytomegalovirus pp65, and so on; Plasmodium falciparum erythrocyte membrane protein (PfEMP1) is the parasitic ligand (35) of NKp30 protein. NKp30 and NKp46 are highly similar in protein expression and signal transduction. When triggering cytotoxicity of NK cells, NKp30 exerts synergistic effects with NKp46 and NKp44. Delahaye et al. transfected NK cells with NKp30a, NKp20b, and NKp30c and found that NKp30b could regulate Th1 cytokine production, and NKp30c played a role in enhancing IL-10 secretion and transmission of inhibitory signals by rapidly phosphorylating p38MAPK (41). In addition, soluble BAG6 has been confirmed to exist in the plasma of patients with CLL, and when CLL develops to the advanced stage, the level of soluble BAG6 in plasma will significantly increase (42). However, exosome BAG6 can enhance the cytotoxicity of NK cells (42). It is a consensus that the anti-tumor effect of NK cells is influenced by tumor microenvironment, and the opposite effect of the same molecule makes the anti-tumor immune mechanism more complicated.

NKp44 protein is expressed on the surface of activated NK cells and acts as a specific marker for activated NK cells. There are three specific ligands for NKp44 protein, which are tumor cell ligands, viral ligands and bacterial ligands. Viral ligands include HA and hn; Bacterial ligands are dominated by cell wall components of Mycobacterium tuberculosis (43). The binding of NKp44 protein to KAPAP/DAP12 is mainly dependent on the lysine residue in the transmembrane region protein. When the two are combined, the ITAM of KAPAP/DAP12 functions as a signaling pathway for activation (44). ITAM undergoes tyrosine residue phosphorylation after NKp44 activation, which is mediated by tyrosine kinase (Syk) and accompanied by phosphorylation of ZAP70. After the activation of this signaling pathway, downstream signal transduction triggers cytotoxicity of NK cells (44).

**Figure 2:**
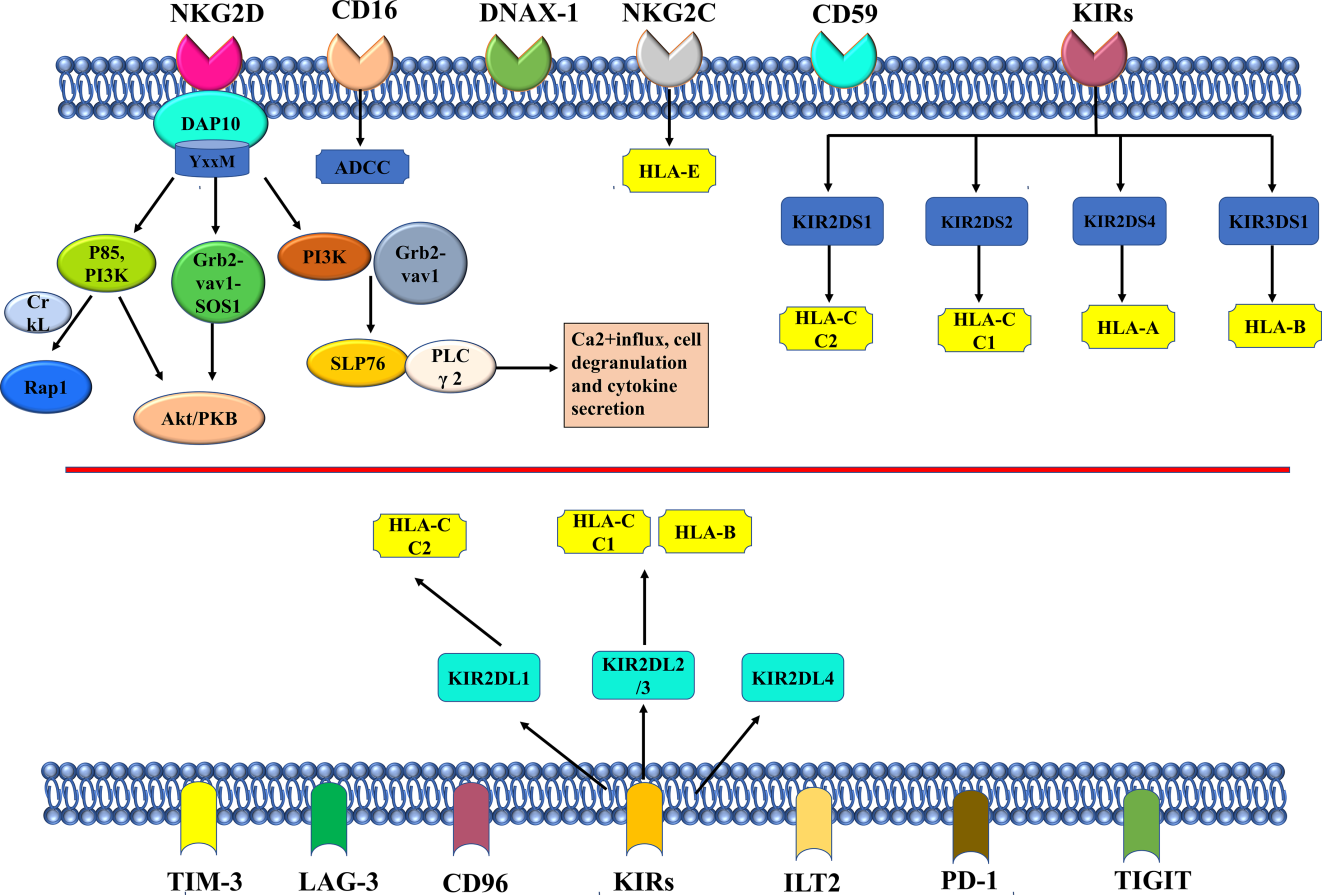
The mechanism of NKG2D, other important activating receptors, and the inhibitory receptors in NK cell.

NKG2D homodimer is another important activating receptor of NK cells. The mechanisms of action for activating receptors such as NKG 2D and inhibiting receptors are summarized in **Figure 2**. The adaptor protein DAP10 can be non-covalently bound to NKG2D, which is an activated receptor in nature. The binding of the two will activate multiple signaling pathways, including mitogen-activated protein kinase MAPK, Janus kinase (Jak)/signal transduction and transcription (STAT) signals, etc. (45). The YxxM motif is the intracellular fragment that DAP10 relies on to bind to p85PI3K, Grb2, and Shc (44). Relevant studies have shown that the crosslinks of DAP10 and NKG2D can further bind to p85 PI3K and Grb2-Vav-1-SOS1 complexes to activate the Akt/PKB signaling pathway (46). Activation of SLP76 and PLC γ 2 generally occurs after PI3K and Grb2-Vav1 complexes are recruited by DAP10 (47). Activation signal finally promotes Ca^2+^influx, cell degranulation and cytokine secretion. Segovis et al. found that the interaction between p85 PI3K and the adaptor protein CrkL is necessary for NK cell activation (48). Small Rap1 belongs to the small Ras family and can be recruited by PI3K and CrkL (48). The protein recognized by NKG2D is highly expressed in tumor cells, but in order to escape the recognition and killing of NK cells, some tumor cells reduce the expression of NKG2D on the surface of NK cells by releasing soluble ligands and secreting immunosuppressive cytokines. Activated receptors of NK cells can work together with receptors of other NK cells to activate cytotoxicity of NK cells. A family of signaling lymphocyte activation molecules including NK-t-b antigen (NTB-A) and 2B4 can activate the cytotoxicity of NK cells by co-stimulation. There is a significant up-regulation of CD48 in EBV-infected B cells and lymphoma cells that is specifically recognized by 2B4 (49). NK cell co-receptors, such as CD5, also play an important role in the process of polarization degranulation (50). CD94/NKG2C heterodimer is composed of type II proteins of the type C lectin family, and both it and killer cell Ig-like receptors (KIRs) belong to the activation receptors of NK cells, which can specifically recognize HLA-I molecules (51). There is limited research on NK cell-activated receptors. According to the results of the current research, as a non-polymorphic MHC class Ib molecule widely expressed in multiple cells, there is a non-monomer peptide binding motif in the HLA-E protein structure that can bind to CD94/NKG2C, but the binding degree is weak (52). The Type I transmembrane molecule KIRs, as a member of the immunoglobulin superfamily, specifically recognizes human leukocyte antigens A, B, and C (HLA-class I) (53, 54). HLA-I molecules are KIRs ligands, but the results of earlier studies supported that the functions of HLA-I and HLA-II molecules in regulating NK cell activity are likely to be closely related to the interaction between NKp44 and HLA-DP, which may be regulated by peptides presented by HLA-DP isoforms (55). Besides, CD16 is another important activating receptor of NK cells which is involved in ADCC (17, 56). CD16 is expressed on large granular lymphocytes (LGLs) and also on granulocytes, tissue macrophages, and subsets of monocytes, eosinophils, and dendritic cells at moderate levels. It has been shown that the CD16 antigen can be non-convalently associated within the membrane of NK cells, and it is widely used in cancer immunotherapy in clinical practice (57, 58).

### Inhibitory Receptors

The inhibitory receptors in NK cells mainly recognize MHC class I molecules which are HLA in humans (59). The inhibitory signaling by MHC-I-specific receptors is essential for hematopoietic cells to avoid destruction by NK cells. The inhibitory receptors of NK cells specific for MHC class I molecules fall into three families, including KIRs, leukocyte immunoglobulin-like receptors (LIRs), and NKG2A.There are five human-derived KIRS, namely, KIR2DL1 that recognizes HLA-C C2 isoforms, KIR2DL2 and KIR2DL3 that recognize HLA-C C1 and two specific HLA-B isoforms, KIR3DL1 that binds to HLA-B and HLA-A isoforms with Bw4 epitopes, and KIR3DL2(56 that recognizes specific HLA-A isoforms (54). After specific binding of KIR2DL, KIR3DL, and CD49/NKG2A to their respective ligands, the ITIM sequence is phosphorylated and activated by SRC family kinase (SFK). The activated ITIM will recruit SHP-1/SHP-2 and down-regulate the phosphorylation level of downstream signaling molecule (XX/YY) that activates the receptor, thereby inhibiting the cytotoxicity of NK cells (60). KIR2DL4 is not a common receptor in humans, because it has both activating and inhibitory signaling domains (61). Ig-like transcript 2 (ILT2) is another HLA-specific inhibitory receptor. NK cells express inhibitory checkpoints that regulate the intensity and range of the immune response, prevent the normal issues from being attacked, and maintain immune cell homeostasis (62). The checkpoints are an important reason for immune tolerance in the genesis and development of tumors (63, 64). The ligands are upregulated on tumor cells. The inhibitory checkpoints of NK cells also include T cell Ig and ITIM domains (TIGIT) and CD96/Tactil.

### Adhesion and polarization of NK cells

**Figure 3:**
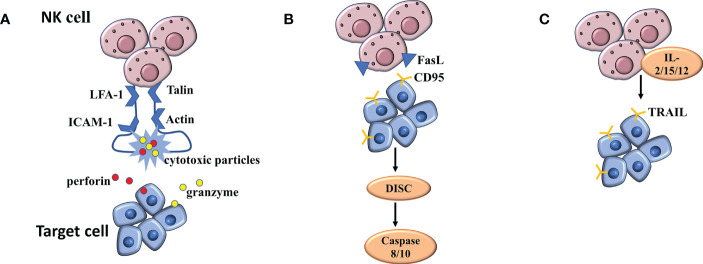
**(A)** The mechanism of NK cell mediated cytotoxicity by releasing cytotoxic particles. **(B, C)** The mechanism of NK cell mediated cytotoxicity binding of NK cells to death receptors.

The functions of NK cells in killing virus-infected cells and tumor cells need to be completed step by step: First, NK cells complete the binding to target cells under the mediation of cell adhesion molecules; Next, NK cells undergo polarization to complete the migration of cytotoxic particles to target cells; Finally, the cell loner releases perforin and granzyme to the target cells (**Figure 3A**). When NK cells exert natural cytotoxicity, the interleukin LFA-1(α L β 2 integrin, CD11a/CD18) plays an important role in mediating the binding of NK cells to target cells. In the LFA-1-deficient mouse model, NK cells were unable to kill the target cells due to impaired coupling formation (65). Similarly, blocking antibodies with LFA-1 can also cause NK cells to lose cytotoxicity to kill target cells (66). A large number of studies have confirmed that NK cells must have the participation of LFA-1 in the process of adhesion to target cells. The cyclic structure formed by LFA-1, talin and actin ensures the stability of the immune synapse formed between NK cells and target cells. This scaffold itself belongs to a special signal complex (67). At the same time, this structure is responsible for the transport of cytotoxic particles and plays a key role in killing the target cells (67). Targeted polarization of microtubule organizing center (MTOC) is a critical link in NK cell killing, followed by accumulation of cytotoxic particles at the immune synapse (67). Abundant MTOC, granular, and microtubule structures are aggregated at the center of the LFA-1-LP actin ring (68). Further studies confirmed that both the initial binding of NK cells to target cells and the reorganization of agonist protein cytoskeleton require the binding of ICAM-1 and LFA-1 to exert the mediating effects, which has a significant cell cycle dependence and is an essential link in particle polarization (69).

### Other mechanisms of NK cell mediated cytotoxicity

The binding of NK cells to death receptors is another mechanism for killing, distinct from exocytosis of dissolved particles. The initiation of apoptotic programs requires the activation of three different receptor/ligand systems: TNF, which binds to TNF receptor -1 or -2, FasL, which binds to CD95(APO-1/Fas) receptor, and TRAIL, which binds to different TRAIL receptors. Regulation of FASL and TRAIL-mediated killing were the next major concerns (**Figures 3B, C**). FASL is a type II transmembrane protein expressed on the surface of activated T cells and NK cells (70, 71). The cells synthesize FASL in the endoplasmic reticulum and transport it to secretory lysosomes through the Golgi apparatus (72). FASL is transported to the surface by degranulation and then rapidly diffuses into the plasma membrane. LFA-1-mediated intrasynaptic adhesion may limit the diffusion of FASL into the plasma membrane (72). In addition, FASL can also be briefly exposed to the surface by incomplete particle fusion (72). The CD95 receptor is locally attached to the surface of virus-infected cells and cancer cells, and FASL, when distributed on the surface, binds to the former, thus activating the apoptotic signaling pathway (73). Subsequently, metalloprotease cleaves the bound FASL, and the resulting soluble FASL is not cytotoxic (74). Under special circumstances, metalloprotease can produce significant inhibition on the apoptotic process caused by FASL in binding state (75). When the CD95 receptor is activated, the internal signaling pathway is stimulated, and the assembly of death induction signaling complex (DISC) occurs, which activates caspase 8 and 10, and induces the final apoptosis through caspase cascade and mitochondrial membrane potential depolarization (76).

Cytotoxic cells such as T cells and NK cells express transmembrane protein II TRAIL(79 that shares homology with FASL and TNF (77). TRAIL was not detected on the surface of freshly isolated NK cells, but surface TRAIL was detected under stimulation by IL-2, IL-15, or IL-12 (78). TRAIL-mediated cytotoxicity is highly dependent upon NK cell killing virus to infect cells and cancer cells, which is common in liver NK cells (79). However, soluble TRAIL is different from soluble FASL in that it maintains apoptotic activity and induces the death of cells around NK cells that secrete TRAIL.

### Mechanism of tumor escape from NK cell immune surveillance

**Figure 4:**
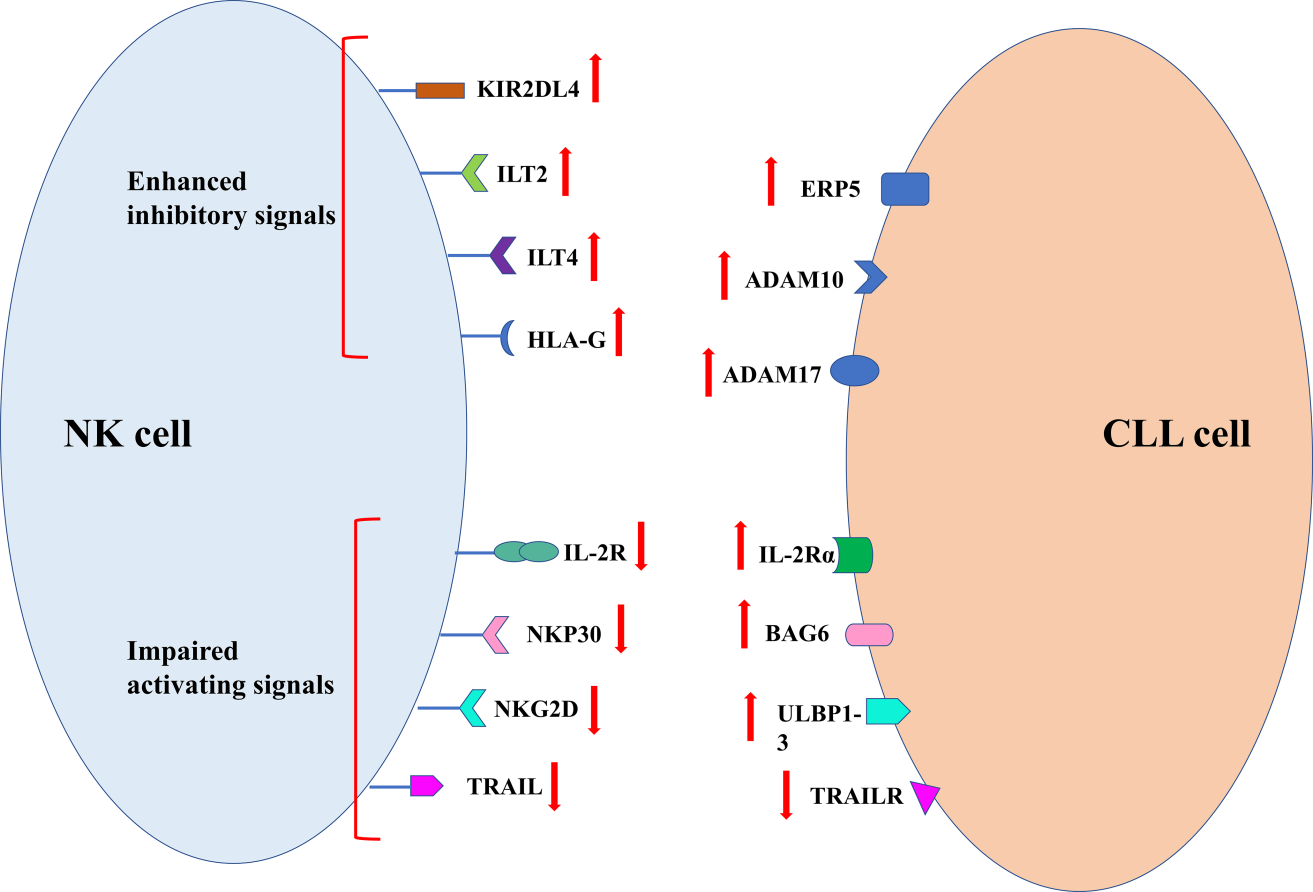
Mechanism of tumor escape from NK cell immune surveillance.

In order to avoid the killing effect of NK cells, tumor cells take corresponding measures. The specific mechanism is summarized in **Figure 4**. The decreased killing effect of NK cells on cancer cells is closely related to this. Clinical studies have shown that NK cell dysfunction is prevalent in a variety of hematological malignancies and solid tumors (80). It is common that up-regulation of inhibitory NK receptor ligands such as KIR2DL4, immunoglobulin-like transcript 2(ILT2) and ILT4 human leukocyte antigen g(HLA-G) in tumor cells is more common in immune stress, whereby cancer cells escape NK cell killing (81). In addition, the poor prognosis in tumor patients was confirmed to be closely related to abnormal HLA-G expression, which most likely helped the tumor to escape from immunotherapy (82). The inhibition of NK cell proliferation and cytotoxicity was attributed to reduced IFN production by HLA-G in combination with IL − T2 − γ and TNF − α (83). The activity of GD2-specific CAR-NK cells can be inhibited by HLA-G expressed on Ewing sarcoma (84). In patients with CLL, blocking HLA-G on tumor cells is considered to be an effective means of sensitizing NK cell immunotherapy (85).

Soluble molecule and ligands produce by tumor cells can also help themselves to escape recognition and killing by NK cells. Soluble IL-2R α produced by tumor cells can bind to IL-2, which is necessary for the proliferation and activation of NK cells. Such competitive binding results in the failure of IL-2R on the surface of NK cells to bind to IL-2, resulting in the reduction of NK cell activity (86). Studies have shown that high expression of soluble ligand of NKp30 and major tissue MHCI related chain A(MICA) in serum and soluble ligand of NKG2D ULBP1-3 in serum can lead to decreased NK cell killing ability (42). Recognition of ligands on tumor cell membrane by NK cells can be blocked by the shedding of soluble ligands in tumor cells (87). Tumor cell shedding ligands require the protein disulfide isomerase ERp5, disintegrant and metalloproteases ADAM10 and ADAM17 to function (87). Ferrari de Andrade et al. confirmed through experiments that the antibodies at the protein cleavage sites of MICA and MICB could inhibit the two proteins from falling off the surface of cancer cells and reactivate the killing effect of NK cells (88).

The binding of NK cells to the death receptor initiates an exogenous apoptotic cascade. The death signal is emitted by the death receptor and transmitted to the cells through a special signaling pathway. The inactivation of the death receptor will cause disorders of the normal apoptotic program, and the above conclusions were verified in metastatic breast cancer samples (89). Mutations of the TRAIL receptor gene are less frequent in human tumors (90–92). The gene mutations of L334F, E326K, E338K and K386N are the tumor-related TRAIL receptor gene mutations discovered so far. These gene mutations can directly lead to the blocked recruitment process of intracellular components such as caspase -8 or FADD, and further cause the loss of TRAIL-R2 function (93). At the same time, the down-regulation of TRAILL-R1 and TRAIL-R2 as well as the apoptosis resistance of B cell malignant tumors are indeed closely related to the 8p chromosome. The common associated alleles in epithelial cancer do suggest poor prognosis and metastasis (94, 95). Similarly, the absence of a functional mutation in the FAS gene in tumor cells helps tumor cells escape CD95L/FasL-binding-activated apoptosis (96). Therefore, tumor cells can resist the killing effect of NK cells through various pathways, regardless of the external or internal apoptotic pathway.

### NK cells in CLL

The NK cells in the patients with CLL were initially found to be impaired in cytolytic activity in the early 1980s, which were believed to result in the NK cell being unable to kill CLL cells and was mainly caused by intrinsic NK cell defects (97, 98). Following findings suggested that immune escape mechanisms of CLL cells were also the cause of impaired cytolytic activity of NK cells apart from intrinsic defects (42, 85). Some other studies reported different results, which showed that the NK cells in peripheral blood of patients with CLL expressed the activity of degranulation, cytokine production and ADCC (99). There are also results indicating that the NK cells in the patients with CLL can be restored with adequate activating signaling from certain types of interleukins (100, 101). Based on those findings, it can be inferred that the NK cells have the potential to play an important part in immunotherapy for the patients with CLL, because their cytolytic activity impairment can be reversed in certain conditions (102). Besides, some later studies find that the low-level expression of HLA-class I molecules is observed in the CLL cells of most patients, and the missing of HLA-class I molecules leads to the killing of tumor cells mediated by NK cells, which also demonstrates the mechanism and effect of NK cells against CLL (103, 104).

It has been found in some studies that the CLL patients have more peripheral NK cells than healthy people, and that the increased amount of NK cells is correlated with better prognosis (105, 106). However, some other studies reported different results about the correlation between the increased amount of NK cells and the prognosis of CLL and did not support the correlation (107). One factor contributing to the different results of the role of NK cells in patients with CLL is CMV infection, in which case the number of mature NK cells expressing more activating receptor CD94/NKG2CO increases adaptively, and the phenotype of the NK cells changes (108). The difference may also come from the study design and subjects of different studies, possibly including the inclusion and exclusion criteria, experiment methods, signaling substances for stimulating the NK cell receptors. According to the findings above, insufficient studies have been conducted on the mechanism and functionality of NK cells in the patients with CLL, so it should be further studied to understand and clarify the effect of NK cells against CLL.

## Immunotherapies involving NK cells in CLL

NK cells are usually involved in the immunotherapies in CLL through stimulated or restored activity of patients’ NK cells or the ones with activity administered to the patients. The so-called adoptive immunotherapy refers to the *in vitro* induction and culture of patients, healthy donors, or autologous or allogeneic NK cells based on NK cell immunotherapy, to exert the effect of directly or indirectly killing tumor cells through these NK cells. The source of NK cells is an important factor in determining the efficacy of this treatment. At present, only autologous NK cells are actually applied to cancer immunotherapy, but NK cells isolated from the peripheral blood of patients are difficult to expand *in vitro* as expected. Studies have confirmed the therapeutic effect of autologous NK cells in digestive system cancer. When the NK cells reached 4720fold amplification *in vitro*, they showed high *in vitro* dissolution activity and strongly expressed functional markers such as NKG2D and CD16, but no clinical response was observed (109). The patient’s treatment likely affected the functional status and proliferative capacity of isolated NK cells *in vitro*, which also explains the poor clinical efficacy of autologous NK cell immunotherapy (110). There is a general problem of high dysfunction and reduced number of NK cells in cancer patients, so it is of great clinical significance to study the adoptive transplantation of allogeneic NK cells. After extensive exploration, Miller et al. found that the effectiveness of allogeneic NK cell adoptive transplantation was confirmed by the results of a clinical experiment of allogeneic NK cell reperfusion. In this experiment, after immunosuppressed AML patients were re-injected with NK cells and treated with IL-2, IL-15, the number of NK cells were increased, and five of the 19 patients were in remission (111). The results of a clinical study by Tonn et al. showed that 75% of the 15 patients with advanced drug-resistant malignancies (lung cancer, leukemia, and lymphoma) developed an anti-tumor response 48h after two injections of NK-92 cells (112). However, whether NK-92 cells can become a new type of cancer treatment still needs further investigation. Studies have proved that NK cells derived from induced pluripotent stem cells (iPSCs) show high cytotoxicity to a variety of tumors *in vivo* and *in vitro* (113). The research by Zeng et al. confirmed that NK cells most likely originated from peripheral blood-induced pluripotent stem cells (114). Next, we will describe in detail the immunotherapy of NK cells in CLL.

### Intensification of NK cell-mediated ADCC

One approach involving NK cells the immunotherapies in CLL works through intensifying NK cell-mediated ADCC, which uses tumor-specific monoclonal antibody (mAbs) or bispecific and trispecific killer engagers (BiKEs or TriKEs) (**Table 1**).

**Table 1:** Tumor-specific antibody therapies.

Target	Antibodies	Anti-tumor effect	Reseaon of limited efficacy	Improving the therapeutic efficacy
CD20	rituximab	complement-dependent cytotoxicity (CDC); direct target cell apoptosis; antibody-dependent phagocytosis; ADCC	the loss of CD20 antigen causing the increase of antigen-loss cells resistant to NK cell-mediated ADCC and reducing the efficacy as a result; poor affinity of rituximab to FcγRIIIa; increase the release of inhibitory substances suppressing the immune reaction through weakening NK cell-mediated ADCC; the impaired NK cell activity in the CLL patients	
	Obinutuzumab			glycoengineering
	Ofatumumab			
	Ublituximab			
CD19	inebilizumab (MEDI-551).			afucosylation: increase the binding affinity of mAbs to FcγRIIIa
				Fc-engineering
CD16	BiKEs(a single-chain variable fragment (scFv) recognizing CD16 + a scFv recognizing tumor antigens)	transferring signals as immunological synapses and to induce cytotoxicity		
	TriKEs	increasing the possibility of identifying tumor cells in the case of one antigen missing and reduces immunological evasion		

#### Monoclonal antibodies

**Figure 5:**
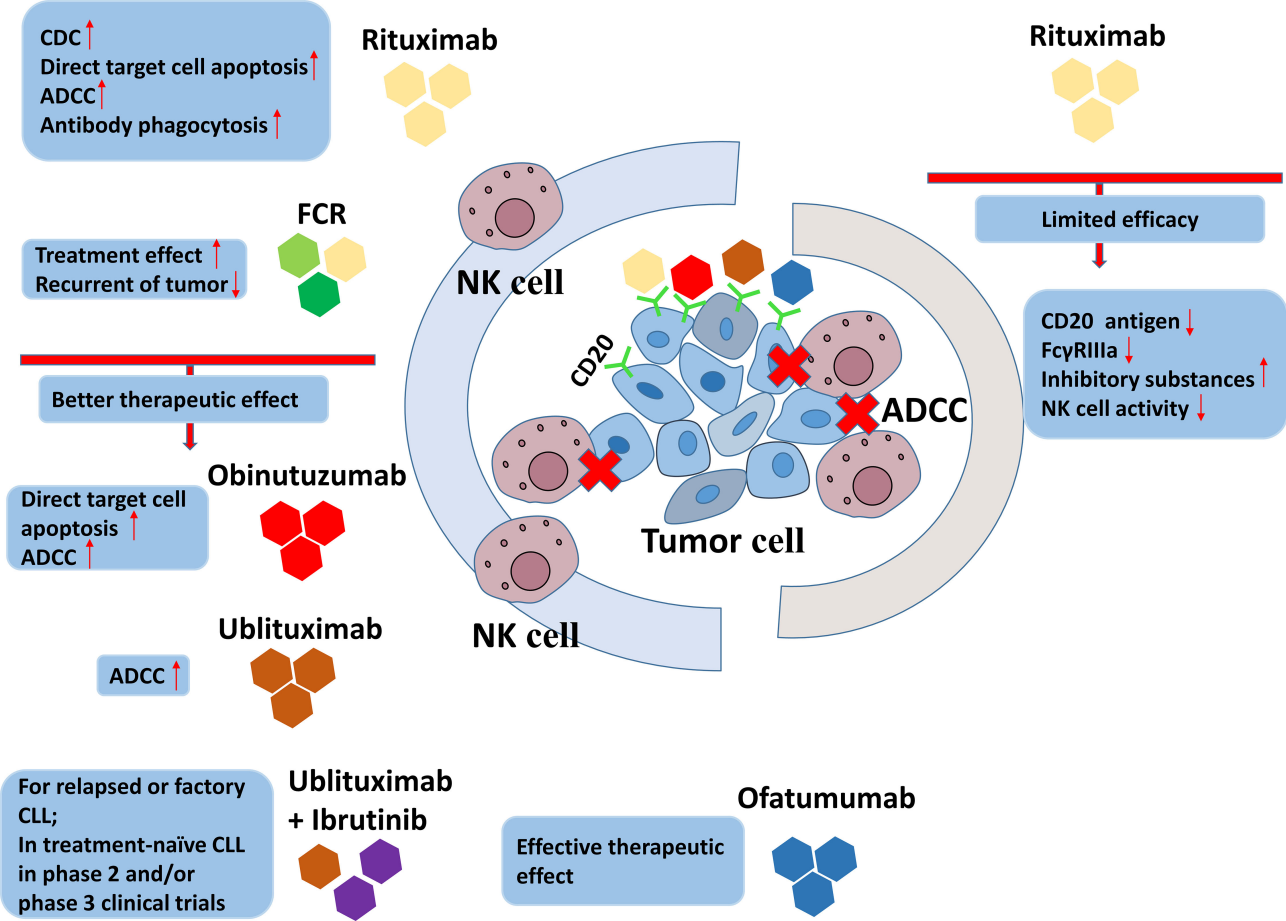
The antitumor mechanisms of anti-CD20 mAbs.

Tumor-specific mAbs are one of the stimuli inducing ADCC medicated by NK cells in immunotherapies in CLL, which recognize the different ligands on the surface of CLL cells, including CD20, CD19 and CD37 (17, 56). The first mAbs employed in immunotherapy in CLL were the family targeting CD20, of which the first approved for clinical use was rituximab (115). All of the antitumor mechanisms of anti-CD20 mAbs are presented in **Figure 5**. Rituximab is approved by FDA for non-Hodgkin’s lymphoma. There are several explanations for the anti-tumor effect of rituximab, including complement-dependent cytotoxicity (CDC), direct target cell apoptosis, antibody-dependent phagocytosis and ADCC (116). Rituximab had low efficacy when used alone, while its joint use of fludarabine and cyclophosphamide (FCR) adds to the efficacy (117). FCR is an option for the patients who have not receive treatment before or those with recurrent tumors (118, 119). The limitations of the use of anti-CD20 mAb alone in CLL immunotherapy are mainly due to the following reasons: First, the CD20 antigen on the surface of CLL cells is lost due to rituximab, which causes an increase in antigen-loss cells that tolerate NK cell-mediated ADCC, resulting in a decrease in efficacy (120). Second, weak binding of FcγRIIIa to rituximab on the surface of NK cells may also result in reduced efficacy (116). Additionally, rituximab can increase the release of inhibitory substances suppressing the immune reaction through weakening NK cell-mediated ADCC (121). The limited therapeutic efficacy of mAbs in immunotherapy of CLL is also possibly associated with the impaired NK cell activity in the CLL patients. To improve the therapeutic efficacy of mAbs in immunotherapy of CLL, glycoengineering were attempted to enhance the ADCC effect to increase the affinity of anti-CD20 mAb to activating Fc receptors. Obinutuzumab was the first humanized mAb modified through being glycoengineered targeting CD20, which was shown to induce stronger ADCC and direct target cell death *in vitro* compared to rituximab (31).

Ofatumumab is a lately approved mAb targeting CD20 at different fragment from rituximab and obinutuzumab (122). Although both ofatumumab and obinutuzumab are proved to be effective in phase 3 clinical trials, ofatumumab is preferred in the later course of therapy than obinutuzumab because it is less affected by the history of treatment (123–125). Ublituximab is a novel glycoengineered anti-CD20 mAb found to induce stronger NK cell-mediated ADCC against CLL cells *in vitro* than rituximab (126). Ublituximab has been shown to have good single-agent activity or efficacy in combination with ibrutinib in relapsed or refractory CLL (127–129).

**Figure 6:**
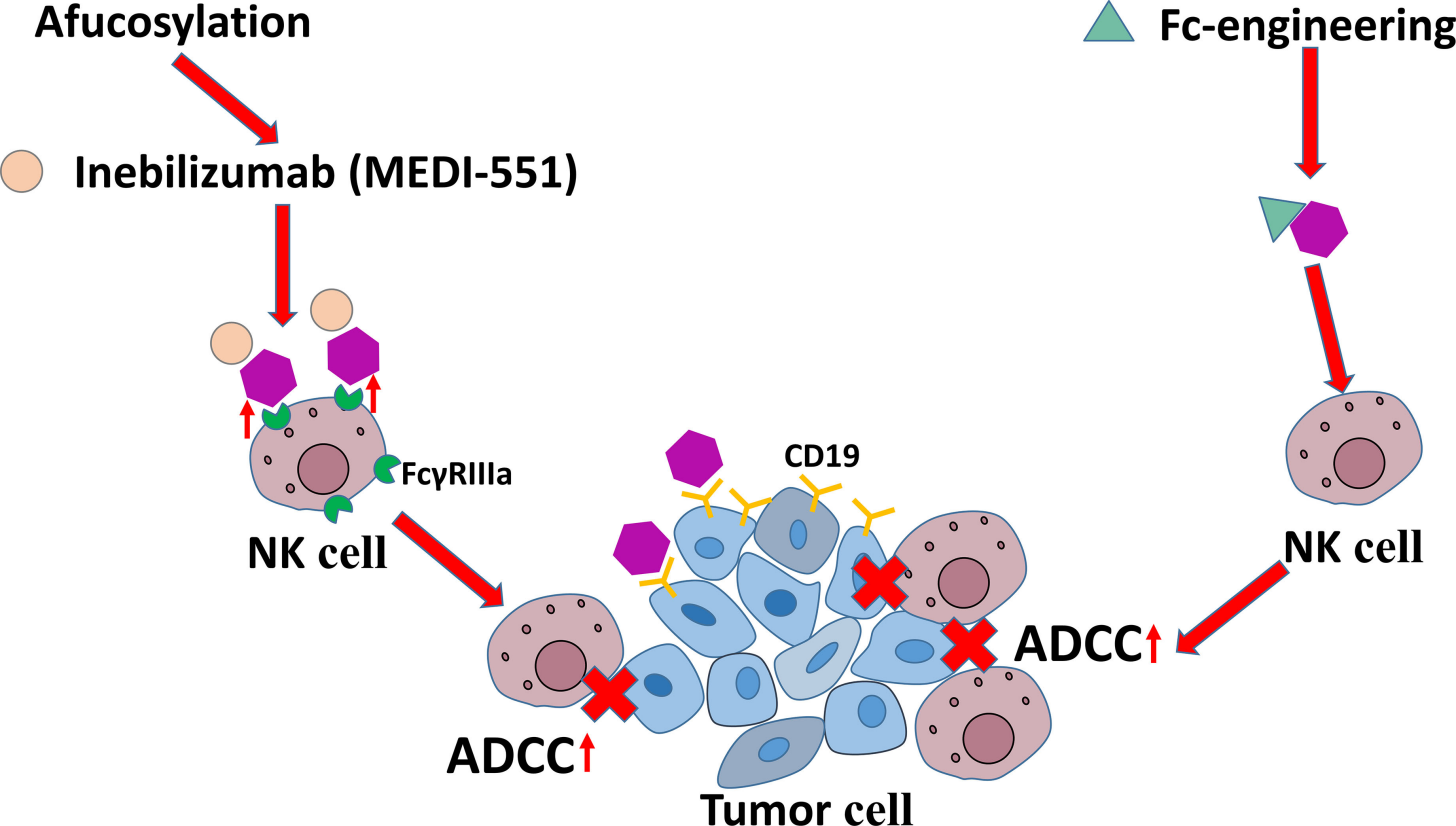
The antitumor mechanisms of anti-CD19 mAbs.

The mAbs targeting CD19 are another family used to intensify NK cell-mediated ADCC. The current roles of anti-CD19 mAbs are shown in **Figure 6**. It has been found that some modifications improve the efficacy of mAbs targeting CD19 in immunotherapies in CLL. For example, afucosylation has been shown to increase the binding affinity of mAbs to FcγRIIIa on NK cells and eventually enhance ADCC potency, which is represented by inebilizumab (MEDI-551). Fc-engineering is also an effective modification to mAb shown to enhance killing effect of NK cells on B lymphoma and leukemia cells (130, 131).

#### Bispecific and trispecific killer cell engagers

BiKEs and TriKEs are additional therapeutic approaches that are newly found to trigger human NK cell effector function through CD16 signaling and induce cytotoxicity and cytokine production (58). BiKEs consist of a single-chain variable fragment (scFv) recognizing CD16 and a scFv recognizing tumor antigens at the same time, which enable them to transfer signals as immunological synapses and to induce cytotoxicity (132). TriKEs recognize two different tumor antigens, which increases the possibility of identifying tumor cells in the case of one antigen missing and reduces immunological evasion compared to BiKEs (133). There are limited studies on therapeutic strategies exploiting BiKEs and TriKEs to trigger NK Cells in CLL, but the results suggest that they are promising in the NK cell immunotherapy in CLL. Feys’ group developed different BiKEs constructs binding CD19 and CD16, and found them able to induce ADCC against primary CLL cells *in vitro* (134, 135). Later, the study of Gleason et al. suggested that a BiKE binding CD16 and CD19 and a TriKE binding CD16, CD19 and CD22 enhanced the NK cell cytotoxicity and production of IFNγ, which increased the efficacy of NK cells in killing CLL cells consequently (136). The TriKE binding CD16, CD19 and CD22 has shown better efficacy in activating NK cells than the agents targeting CD20 with higher expression than CD19, which means the combined stimulation of CD22 and CD19 is a better option (136). A TriKE recognizing CD16, CD19 and IL-15 cytokine can activate the NK cells from healthy donors and cause killing of CLL cells, showing promising efficacy in refractory CLL (137).

### Allogeneic NK cell therapy

Before BCR and Bcl-2 inhibitors were found to be effective and started to be employed in treating high-risk CLL, allogeneic hematopoietic stem cell transplantation (allo-HSCT) was the only curative option for the patients with recurrent or refractory CLL (138). The introduction of BCR and Bcl-2 inhibitors in CLL has given rise to the concerns of the risks and benefits of allo-HSCT in high-risk CLL, which greatly decreased the application of allo-HSCTs (139, 140).

In haplo-HSCT for high-risk lymphoid leukemia, the KIRs on the NK cells from the donors do not bind to the HLA on the surface of the patients’ leukemia cells, so the NK cells do not receive inhibitory signaling from the binding of KIRs and HLA and therefore get activated and start to kill leukemia cells. Bases on this mechanism, allogeneic NK cells from the donors are very important in graft-versus-leukemia (GvL) effect, which has been adequately studied and proved (141, 142). Besides, *in vitro* studies have found evidence on the ability of allogeneic NK cells to kill CLL cells without recourse to KIR–HLA mismatches (143). On the contrary, the G*v*L effect in allo-HSCT for CLL relies primarily on T cells (144, 145). However, the GvL effect mediated by T cells may lead to graft-versus-host disease (G*v*HD) in HSCT for CLL. By contrast, allogenic NK cells can be a safer option for immunotherapy for CLL because they do not mediate GvL effect directly (141, 142, 146).

### CAR-NK cell therapy

At present, CAR T cell therapy is one of the focuses of the adoptive immunotherapies, and its application in acute lymphoblastic leukemia, CLL, or lymphoma has achieved good outcome in clinical practice. For example, anti-CD19 CAR T cells are already approved for clinical use in the patients with aggressive B-cell NHL or acute lymphoblastic leukemia.

CAR-NK cell therapy has become an emerging field of adoptive immunotherapies, and increasing number of pre-clinical studies have been conducted on it. CAR-NK cell therapy has shown the potential to be a good option comparable and even superior to CAR T cell therapy, because NK cells can be activated without antigen-presenting and kill a broad range of tumor cells (147). Besides, NK cells provided an extra mechanism of killing tumor cells by ADCC on the basis of CAR-mediated cell lysis (148). Another important advantage of CAR-NK cell therapy is that it does not require complete HLA-matching for safe use, so one modification can apply to multiple patients, which may save the waiting time for CAR-NK cell production and reduce the price of the therapy (149). Additionally, NK cells are safer for not causing GvHD while taking effect (150). Due to the above advantages, NK cells are being widely studied to explore their application in adoptive immunotherapy (151). We summarize some clinical trials related to NK cell based CLL therapy currently in progress in **Table 2**, with data from https://clinicaltrials.gov/.

**Table 2:** Current NK-cell immunotherapy clinical trials for CLL.

Identifier	Source of NK cells	Phase	Treatment method design
NCT05020678	Allogeneic	1	NKX019
NCT04796688	AT19	1	CAR-NK-CD19 + Fludarabine and Cyclophosphamide
NCT03056339	Cord blood derived	1	iC9/CAR.19/IL15-transduced NK cells + Fludarabine, Cyclophosphamide or Mesna
NCT04848064	Allogeneic	1	NK cell infusion + Fludarabine, Cyclophosphamide and Mogamulizumab
NCT02727803	NK92	2	NK92 + cord blood transplant + Chemo + Rituximab
NCT02890758	Non-HLA matched donor	1	NK cells + ALT803

NK cells are easily acquired from peripheral blood, umbilical cord blood and other sources, which makes it possible for the wide use in clinical practice (152). The CAR NK cells derived from cord blood attract more attention now, because they are available, easily proliferated, and possible for better activation profile (152). Preclinical studies have shown the efficacy of CAR NK cells derived from cord blood in defeating CLL cells, and their safety and effectiveness is being further explored in several clinical trials (153). One of the clinical trials shows that CAR NK cells derived from cord blood targeting CD19 are safe without causing major adverse effects including cytokine release syndrome, neurotoxicity, and GvHD (154). However, the perdurability of CAR NK cells therapy is not fully tested *in vivo* in this trial because the interference of other therapies after 30 days of experiment.

The individualized preparation process of CAR-T cells is expensive. However, CAR NK cells can be prepared into “off the shelf” products without being restricted by autologous cells, because they are safe enough and rarely cause GVHD during allogeneic infusion. NK cells usually secrete a limited level of IFN- γ and GM-CSF, but does not secrete the major cytokines IL-1 and IL-6 that trigger cytokine celease syndrome. In addition, CAR NK cells express natural recognition receptors. These receptors can recognize CAR independent stress-induced ligands, thus reducing the possibility of disease recurrence associated with the loss of CAR targeted antigens. In view of the above advantages of NK cells, researchers are currently exploring the application of CAR NK cells in the treatment of blood and solid tumors, with broad prospects. Nevertheless, there are still some factors affecting the clinical transformation of CAR NK cells. Unlike T cells, arming NK cells with CAR is challenging, because NK cells from peripheral blood have low transfection rate and short survival time *in vivo* (114). Many researchers are exploring ways to improve the transfection efficiency of primary NK cells, but no significant progress has been made. In addition, the activation of CAR-NK cells is affected by the location of CAR-binding epitope. The distance between the surface of CAR-NK cells and CAR-binding epitope is also a factor. Contamination of allogeneic NK cells by T-cells can induce GvHD or lymphoproliferative disorders (155).

In summary, NK cell-based immunotherapy is a valid alternative of the therapeutic treatment for the patients with CLL according to the current evidences.

## Conclusions

NK cells have become a promising and advantageous approach for the immunotherapies of CLL with the different mechanisms against leukemia cells from earlier treatment. NK cell-based immunotherapy not only provides a second choice for the leukemia types resistant to routine treatment, but also reduces immune escape through motivating the potential of the patients’ own immune system. The efficacy of NK cell-based immunotherapy is being evaluated by more and more clinical trials. The research on the NK cell receptors, including 2B4, CS1 and LLT1, and their ligands may help further clarify the mechanisms of leukemia and improve the therapies. Although previous evidences have demonstrated the efficacy of NK cells used as a single agent in adoptive immunotherapy or in tumor-specific mAbs treatment, the proliferation and persistence enhancement of such NK cells *in vivo* can be contributed by the combination effects of cytokines, e.g. IL-12, IL-15 and IL-21.

Although few studies have been conducted to design CAR structures based on the characteristics of NK cells, their killing activity can be increased by CAR powerfully. Thus, most CAR-NK cells simply follow the design of CAR-t cells without taking into account the specific characteristics of NK cells. Designing optimized CAR structures suitable for NK cells and then transfecting the CAR into memories like NK cells or specific NK cell subsets is a promising research direction. Kaufman and colleagues recently established and compared nine different NK cell-specific CAR constructs. To prepare mesoleptically targeted CAR NK cells, different NK cell-specific activation domains and human iPSC were applied. NK specific CAR-NK cells, especially the optimized NKG2D-2B4 ζ- IPSC NK cells, significantly enhanced the killing ability, significantly inhibited tumor growth and prolonged survival time in ovarian cancer xenotransplantation models. In addition, the immune evasion of leukemia cells is also a focus of future studies, which may provide more information on the associated microenvironment and reference for optimizing the therapies.

## Author contributions

HD and FH were involved in the conception, design, analysis, and interpretation of the data. Z-HW and WL were involved in drafting the paper and revising it critically for intellectual content. All authors contributed to the article and approved the submitted version.
